# UNIQUE MICROBIOME SIGNATURES RELATED TO CANCER SURVIVORSHIP AND COGNITIVE DECLINE IN OLDER ADULTS

**DOI:** 10.1093/geroni/igad104.2240

**Published:** 2023-12-21

**Authors:** Jayden Haggler, Brandi Miller, Diptaraj Chaudhari, Hariom Yadav, Shalini Jain

**Affiliations:** University of South Florida, Tampa, Florida, United States; University of South Florida, Tampa, Florida, United States; University of South Florida, Tampa, Florida, United States; University of South Florida, Tampa, Florida, United States; University of South Florida, Tampa, Florida, United States

## Abstract

Significant scientific advancements have resulted in new and effective therapies for patients with cancer. Although this leads to an increased cancer survivor population, there are several long-term side effects of cancer treatments, including a high risk of developing dementia. Emerging evidence shows that the gut microbiota significantly contributes to brain health and dementia pathology; however, its role in cancer treatment-related side effects, specifically in older adults, is not well known. Here, we used 110 participants from our MiaGB (Microbiome in aging Gut and Brain) consortium aged 60 years and older and analyzed their gut microbiome (using whole genome sequencing) with cognitive function (using Montreal Cognitive Assessment (MoCA) and MiniCog). We observed an 8% increase in cognitive impairment (CI) in cancer survivors compared to non-cancer participants (46% vs 38%, respectively). When only comparing cognitively healthy versus CI, the abundance of Escherichia coli was significantly higher in the CI group, while Streptococcus thermophilus was higher in the cognitively healthy group. Additionally, the abundances of Dorea sp. CAG 317, Ruminococcus gnavus, Roseburia faecis, and Eggerthella lenta were significantly increased in the gut of cancer survivors compared to non-cancer participants. Specifically, the abundance of Dorea sp. CAG 317 was increased in cancer survivors with CI. These findings suggest that unique microbiome signatures induced by cancer treatments may impair cognitive function. Furthermore, understanding the associations between the microbiota and cognitive function in cancer survivors will aid the development of therapeutics to combat cancer treatment-related side effects, which are a growing public health concern.

